# High species diversity and turnover in granite inselberg floras highlight the need for a conservation strategy protecting many outcrops

**DOI:** 10.1002/ece3.5318

**Published:** 2019-06-13

**Authors:** Colin J. Yates, Todd Robinson, Grant W. Wardell‐Johnson, Gunnar Keppel, Stephen D. Hopper, Antonius G. T. Schut, Margaret Byrne

**Affiliations:** ^1^ Department of Biodiversity Conservation and Attractions Biodiversity and Conservation Science Kensington Western Australia Australia; ^2^ School of Earth and Planetary Sciences Curtin University Perth Western Australia Australia; ^3^ School of Molecular and Life Sciences, Centre for Mine Site Restoration Curtin University Perth Western Australia Australia; ^4^ School of Natural and Built Environments, Natural and Built Environments Research Centre University of South Australia Adelaide South Australia Australia; ^5^ Biodiversity, Macroecology and Conservation Biogeography Group, Faculty of Forest Sciences and Forest Ecology University of Goettingen Gőttingen Germany; ^6^ School of Plant Biology, Centre of Excellence in Natural Resource Management The University of Western Australia Albany Western Australia Australia; ^7^ Plant Production Systems Wageningen University Wageningen The Netherlands

**Keywords:** beta diversity, conservation strategy, generalized dissimilarity modeling, granite inselbergs, OCBIL theory, rock outcrops, species turnover, water availability

## Abstract

Determining patterns of plant diversity on granite inselbergs is an important task for conservation biogeography due to mounting threats. However, beyond the tropics there are relatively few quantitative studies of floristic diversity, or consideration of these patterns and their environmental, biogeographic, and historical correlates for conservation. We sought to contribute broader understanding of global patterns of species diversity on granite inselbergs and inform biodiversity conservation in the globally significant Southwest Australian Floristic Region (SWAFR). We surveyed floristics from 16 inselbergs (478 plots) across the climate gradient of the SWAFR stratified into three major habitats on each outcrop. We recorded 1,060 species from 92 families. At the plot level, local soil and topographic variables affecting aridity were correlated with species richness in herbaceous (HO) and woody vegetation (WO) of soil‐filled depressions, but not in woody vegetation on deeper soils at the base of outcrops (WOB). At the outcrop level, bioclimatic variables affecting aridity were correlated with species richness in two habitats (WO and WOB) but, contrary to predictions from island biogeography, were not correlated with inselberg area and isolation in any of the three habitats. Species turnover in each of the three habitats was also influenced by aridity, being correlated with bioclimatic variables and with interplot geographic distance, and for HO and WO habitats with local site variables. At the outcrop level, species replacement was the dominant component of species turnover in each of the three habitats, consistent with expectations for long‐term stable landscapes. Our results therefore highlight high species diversity and turnover associated with granite outcrop flora. Hence, effective conservation strategies will need to focus on protecting multiple inselbergs across the entire climate gradient of the region.

## 1 INTRODUCTION

Patchily distributed outcrops of granite occurring as dome‐shaped hills or low mountains, disk‐like pavements, and block‐ or boulder‐strewn hills are found on the crystalline shields of all continents across a wide range of climates and biomes (Migon, 2006; Porembski & Barthlott, 2000; Twidale & Vidal Romaní, 2005). These landforms are relict features, often of great antiquity, that have maintained relief as the adjacent surrounds have been reduced by erosion to expose bedrock (Bremer & Sander, 2000; Campbell, 1997). Typically, they have sharply defined boundaries where ecological conditions and plant assemblages differ moderately to strongly with their surrounds (McGann, 2002; Parmentier, Stévart, & Hardy, 2005; Porembski, Seine, & Barthlott, 2000). Because of these characteristics, granite outcrops are regarded as habitat islands (Ornduff, 1987) and are often referred to collectively as inselbergs (island hills; Bornhardt, 1900).

Generally, environmental conditions on granite inselbergs are stressful for plant life (Szarzynsnki, 2000) and can change markedly over relatively short distances (Schut et al., 2014), providing heterogeneity that supports plant diversity. On summits and gentle inclines, numerous depressions of varying size and depth occur where soil has accumulated. The soils are shallow and porous, and environmental conditions vary seasonally from dry, with high temperatures and evapotranspiration, to humid or waterlogged. These soil patches are isolated from one another by more or less‐extensive areas of bare rock, and vegetation is generally herbaceous or low shrubland. In contrast, at the base of inselbergs, run‐off water from rock sheets upslope can accumulate in areas where deeper soils have developed. Here, vegetation is more continuous and may be denser and taller than on the inselberg, or indeed in the surrounding landscape (Burke, 2002; Porembski et al., 2000; Schut et al., 2014). Thus, island biogeography theory (McArthur & Wilson, 1967) and the influence that island area and isolation have on species diversity may apply to granite inselbergs as isolated features (Porembski et al., 2000).

To date, most studies of plant diversity on granite inselbergs have focused on the putative role of environmental and stochastic factors influenced by dispersal ability and island biogeographic factors such as inselberg area and isolation, and less on geoclimatic history. Studies of granite inselberg vegetation elsewhere show that variation in species richness and composition is partly correlated with environmental variation, particularly soil depth, topographic, and regional climate gradients all of which affect water availability (McGann, 2002; Parmentier et al., 2005; Sarthou, Pavoine, Gasc, Massary, & Ponge, 2017); partly with dispersal abilities of species (Parmentier et al., 2005); and partly with geographical features such as inselberg area and isolation through their postulated influences on extinction and colonization (McArthur & Wilson, 1967; Porembski et al., 2000). However, patterns of species richness and composition on granite inselbergs may also be an outcome of historical processes arising from speciation, extinction, and range dynamics under past environmental conditions and landscape connectivity (Hopper, 2009).

Granite inselbergs are found in local regions across the breadth of Australia (Bayly, 2011; Hopper, 2000). They are especially common and conspicuous in the flat terrain of the Southwest Australian Floristic Region (SWAFR sensu Gioia & Hopper, 2017). As in other parts of the world, the conditions on Australian inselbergs and their isolation from similar habitats have fostered the evolution of endemic plant species (Hopper, Brown, & Marchant, 1997; Hunter & Clarke, 1998; McGann, 2002). Furthermore, because environments on or around granite inselbergs contain both water gaining and shedding sites, they can extend the ranges of mesic species at arid margins and arid species at mesic margins (Hopper et al., 1997; McGann, 2002). Consequently, floras on and immediately surrounding inselbergs make significant contributions to regional species diversity and are important focal points for conservation. By way of example, granite inselbergs in the SWAFR occupy less than 1% of the land area but support *ca*. 17% of its vascular native flora (Hopper et al., 1997).

Due to their ancient origin and relative climatic stability, granite outcrops in the SWAFR can be considered old climatically buffered landscapes or OCBILs (Hopper, 2009). These circumstances may have reduced the intensity of extinctions associated with Pleistocene climate cycles, favored persistence rather than seed dispersal, and promoted the accumulation of species richness by encouraging genetic divergence between populations (Hopper, 2009). Studies of OCBIL inselberg systems on other geology types show that geoclimatic history is a strong organizing force in community assembly (Zappi, Moro, Meagher, & Lughada, 2017). Their closest analogues are granite inselbergs (also OCBILS) in the Mediterranean climate and megadiverse Cape Floristic Region (CFR) of South Africa, but as with the SWAFR granites there are few published quantitative studies of their plant diversity.

Determining patterns of plant diversity on granite inselbergs together with their environmental, biogeographical, and spatial correlates is an important task for conservation biogeography globally due to mounting threats which include mining, water harvesting, urbanization, grazing, and weed invasion (Porembski et al., 2016). Here, we focus on SWAFR inselbergs because they are distinct features in a region that is a centre of plant diversity and endemism, and an internationally recognized biodiversity hot spot (Gioia & Hopper, 2017; Myers, Mittermeier, Mittermeier, Fonseca, & Kent, 2000). However, to date there have been no quantitative investigations of floristic diversity for the region's granite inselbergs. This information will assist conservation planning and management by enabling assessment of the relative conservation significance of individual inselbergs, and of inselbergs collectively in the region. Of specific interest is identifying areas of high species richness and patterns of species turnover.

The beta diversity of granite inselberg vegetation across environmental gradients may reflect two different phenomena, spatial species turnover and nestedness of species assemblages, which result from opposite processes of species replacement and species loss, respectively (Baselga, 2010). Spatial species turnover between sites arises when some species are replaced by others due to niche differentiation or to spatial and historical constraints on connectivity and dispersal. Contrary to spatial species turnover, nestedness occurs when the species assemblages at sites with smaller numbers of species are subsets of the assemblages at richer sites, reflecting a nonrandom process of species loss (Baselga, 2010). The distinction between spatial turnover and nestedness is important for conservation because they require different strategies (Baselga, 2010). Where species turnover is due mainly to species replacement, conserving many different sites, not necessarily a few of the richest ones, is the most appropriate strategy (Angeler, 2013; Baselga, 2010). Where species turnover is due mainly to nestedness, prioritizing a subset of the sites richest in species is considered an adequate conservation strategy (Baselga, 2010).

High rates of beta diversity are reported for granite inselberg plant communities (e.g., Porembski, Martinelli, Ohlemuller, & Barthlott, 1998; Porembski et al., 2000). However, there have been no investigations of the contributions that spatial species turnover and nestedness make to spatial variation in floristic composition. Narrow geographic ranges and species replacement series (e.g., *Borya*) are common in granite‐endemic or granite‐centered taxa (Churchill, 1987) in the SWAFR and, while environmental factors have a strong influence on where species will grow, stochastic factors influencing colonization and extinction over very long time frames may also have a large influence on whether they are present at suitable sites (Porembski et al., 1998, 2000; Yates, Hopper, Brown, & Leeuwen, 2003; Yates, Ladd, Coates, & McArthur, 2007).

Here, we undertake an investigation of three plant communities that occur in different habitats on granite inselbergs in the Mediterranean climate region of the SWAFR. Our aims are to quantify for each community: (a) the patterns of diversity and correlates of species richness in each of the three habitats at the plot and outcrop level; (b) the relative importance of environmental dissimilarity and geographical distance with species turnover in each of the three habitats by partitioning their variance; and (c) the contributions of spatial species turnover and nestedness to their compositional dissimilarity. We discuss the implications of our findings for conservation.

We expect that species richness and variation in floristic composition will be correlated with local‐ and regional‐scale environmental and spatial variables. We hypothesize that the compositional dissimilarity (beta diversity) of granite inselberg floras will increase with environmental and geographic distance. We also expect that species replacement manifested as spatial species turnover, and not nestedness, will be responsible for differences in compositional dissimilarity, based on the predictions of Hopper (2009) for OCBILs. This would advocate a conservation strategy that protects multiple sites, as first articulated in the 1970s as part of the SLOSS debate (single large v/s several small reserves—Diamond, 1975; Lindenmayer, Wood, McBurney, Blair, & Banks, 2015; Wilson & Willis, 1975) and applied in the SWAFR from population genetic studies of *Eucalyptus caesia* and other species in the 1980s (Moran & Hopper, 1983, 1987).

## 2 METHODS

### 2.1 Study area

In Australia, granite inselbergs are common in the SWAFR, occurring on the ancient Yilgarn Craton and Albany‐Fraser Orogen (Myers, 1997). The geologically oldest inselbergs occur in the southwest and form part of the Yilgarn Craton (2,700–2,600 My old). To the south of the Yilgarn, inselbergs also occur on the younger (1,700–1,200 My old) Albany‐Fraser Orogen. Compared with many other floras, especially those of Europe and North America, the flora of the SWAFR has persisted for an extremely long period without any major extinction episodes associated with glaciation, vulcanism, or mountain building (Hopper & Gioia, 2004). Some granite inselbergs may have had their summits exposed since the mid‐Cretaceous, suggesting that opportunities for continuous terrestrial evolution have existed for an extraordinarily long time (Hopper, 2009; Watchman & Twidale, 2002).

**Figure 1:**
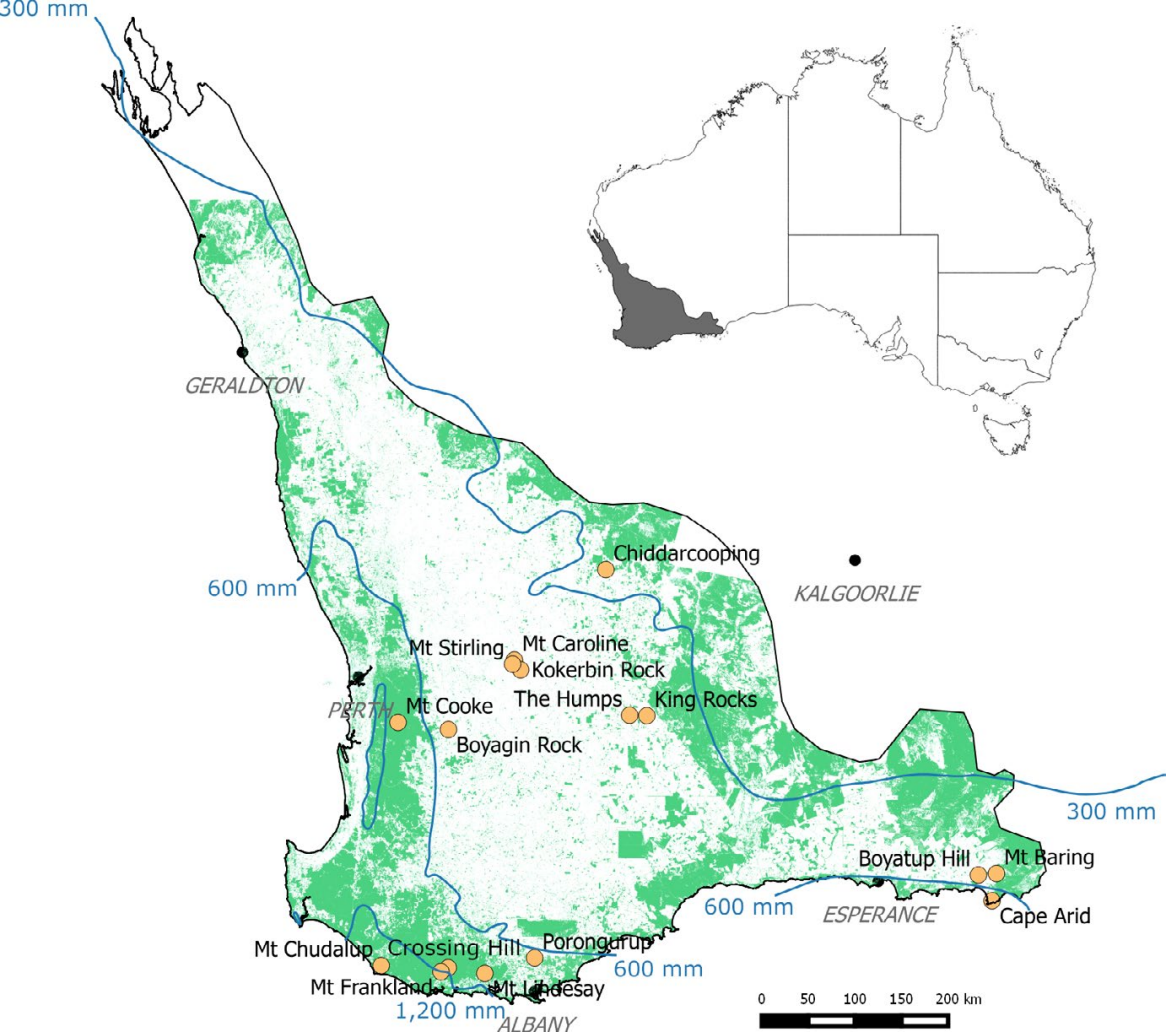
Location of the Southwest Australian Floristic Region and the 16 granite inselbergs surveyed for floristic composition with average annual rainfall isohyets in blue lines and remnant native vegetation shown as green areas

The SWAFR is a relatively wet to semiarid geographically isolated continental refuge *ca*. 300,000 km^2^ in area (Gioia & Hopper, 2017). The region currently has a Mediterranean‐type climate with most rain falling in the winter months May to September. The length of the summer dry season increases with decreasing mean annual rainfall (MAR). Gradients of decreasing rainfall and increasing temperature extend from the west and south coast inland (Figure 1). We sampled the floristics of 16 granite inselbergs, which are typical of the SWAFR, ensuring inclusion of large and iconic inselbergs, and incorporating the climatic gradient from high (>600 mm) to transitional (300–600 mm) MAR zones (Hopper, 1979), taking in gradients of the southern and west coasts of the region. Thus, granite inselbergs were sampled in areas receiving *ca*. 300–1,400 mm MAR. The greatest distance between our study inselbergs was 670 km and the shortest 5 km (Figure 1, Table 1, Schut et al., 2014).

**Table 1:** Characteristics of the 16 studied granite inselbergs in the Southwest Australian Floristic Region

Outcrop	Latitude	Longitude	No. of plots per inselberg	Area (ha)	Isolation (no. of inselbergs within a 20 km radius)	Mean temperature wettest quarter (°C)	Mean temperature driest quarter (°C)	Precipitation wettest quarter (mm)	Precipitation driest quarter (mm)
Cape Arid	123.216203	−33.976505	29	808	3	12.7	20.1	250.6	39.9
Mt Baring	123.248087	−33.710928	30	120	3	11.6	21.1	206.6	34.5
Boyatup Hill	123.041089	−33.735316	30	39	6	11.8	20.9	209.8	34.6
Boyagin Rock	116.881743	−32.470557	31	85	9	11.5	22.9	262.1	11.7
Chiddarcooping	118.658884	−30.905332	30	1,072	7	22.7	25.6	129.3	12.7
Mt Caroline	117.633414	−31.792735	30	240	7	21.9	24.7	135.4	8.3
Crossing Hill	116.875488	−34.784312	25	62	6	11.4	19.4	554.0	69.6
Mt Cooke	116.303876	−32.398995	30	726	9	11.8	22.5	624.4	20.9
Kokerbin Rock	117.704666	−31.887499	30	50	6	21.5	24.3	139.6	9.5
King Rocks	119.152278	−32.317020	30	422	5	21.0	23.7	143.4	11.2
Mt Lindesay	117.307131	−34.839699	24	1822	11	11.5	19.4	434.6	78.9
Mt Chudalup	116.085762	−34.760500	36	118	4	11.8	19.1	732.6	53.9
Mt Frankland	116.790109	−34.824122	33	94	10	11.7	19.5	606.8	71.1
Porongurup	117.892454	−34.686191	30	1810	16	10.1	18.7	255.5	65.3
Mt Stirling	117.612366	−31.833241	30	311	7	21.7	24.5	133.4	8.4
The Humps	118.955721	−32.315829	30	173	10	21.0	23.7	154.4	10.3

### 2.2 Floristic data

**Figure 2:**
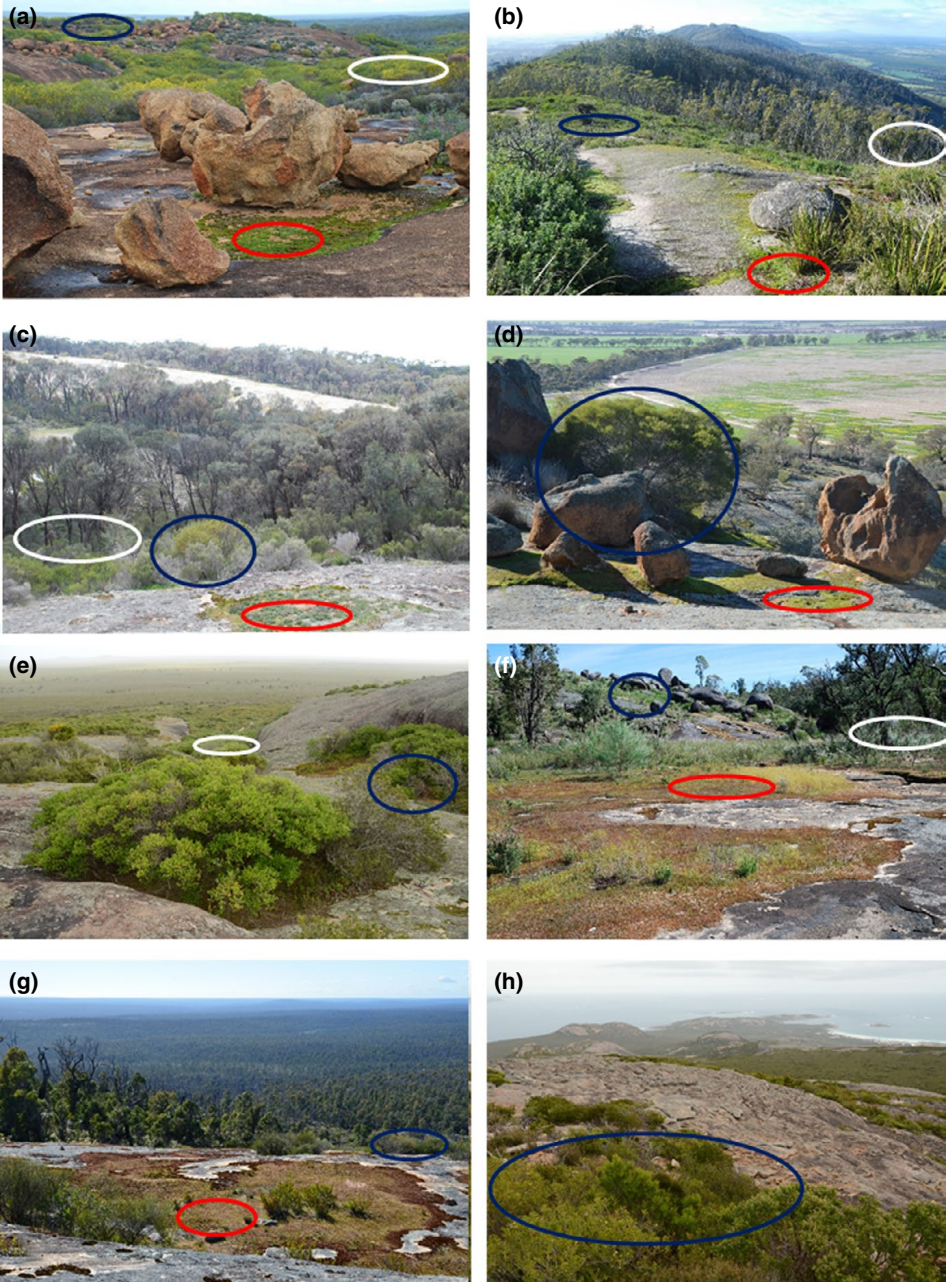
Granite inselbergs in the South West Australian Floristic Region, showing habitats referred to in the text (herbaceous vegetation of soil‐filled depressions on the outcrop (HO)—red outlines, woody vegetation of soil‐filled depressions on the outcrop (WO)—blue outlines and woody vegetation on deeper soils at the base of the outcrop (WOB)—white outlines). (a) Semiarid Chiddacooping, a low relief inselberg in the Transitional Rainfall Zone; (b) large granite range—Porongurup (max alt 650 m); (c) lower slopes of Boyagin Rock, showing *Allocasuarina huegeli* (rock sheoak) bordering a streamline running off the rock; (d) Mount Caroline, a granite outcrop surrounded by cleared vegetation in the Western Australian wheatbelt; (e) near coastal Boyatup Hill in the Transitional Rainfall Zone; (f) lower slope Mount Cooke in the High Rainfall Zone; (g) upper slopes of Mt Cooke; (h) Coastal Mount Arid in the Transitional Rainfall Zone

We chose 16 granite inselbergs based on geographic representation across the region's rainfall gradients. We stratified each inselberg into three major habitat types: herbaceous vegetation of soil‐filled depressions on the outcrop (HO); woody vegetation of soil‐filled depressions on the outcrop (WO); and woody vegetation on deeper soils fringing the base of outcrops (WOB; Figure 2). We included habitats fringing the base of outcrops because of their importance for extending the geographic ranges of species found in other environments and because there are numerous granite‐endemic or granite‐centered species that are restricted to this zone (Hopper et al., 1997). Within the three habitat types, we established up to 14 plots, at locations spread across each inselberg to sample as much of the topographic variation as possible (HO 8–14; WO 10–12; WOB 10; 478 in total). The size of plots varied between the three habitats (i.e., 1 × 1 m for HO; 5 × 5 m for WO; 20 × 20 m for WOB plots).The different plot sizes were based on the size of the dominant species in each habitat (e.g., herb in HO v shrub in WO v tree in WOB) and are suitable for our aims because we do not compare diversity among the three habitat types. Plot coordinates were collected using a GNSS receiver (Garmin Etrex 10).

In the Australian spring of 2010 and 2011, we recorded the presence of all vascular plants in the 478 plots. We also compiled comprehensive field herbaria for each inselberg and collected specimens of each recorded taxon for identification by botanists at the Western Australian Herbarium. In plant groups where taxonomy is under major revision (e.g., *Lepidosperma*), or that are diverse and/or difficult for nonexperts to identify (e.g., Orchidaceae, Stylidiaceae), identifications were undertaken or confirmed by botanists with specialist expertise. Species’ nomenclature followed current usage at the Western Australian Herbarium.

### 2.3 Environmental data inselberg level

The spatial extents of the 16 inselbergs were delineated in ArcGIS v 10.4 (ESRI, 2015) through interpretation of corporate orthophotos (2007–2012, 0.5‐m resolution) and observation of high‐resolution DEM‐derived (see below) contour lines. We also used Google Earth with elevation exaggeration to assist with visualization of the rocky formation in the landscape. We used the predominance of exposed granite, change in relative elevation, and the individuality of the formation in the landscape to define the perimeter of the inselberg. The subsequent polygon was our measure of inselberg area and included all HO and WO plots and most WOB plots fringing the granite. Some WOB plots were located in drainage lines beneath the granite inselberg and were adjacent to the polygon.

The isolation of each of the 16 study inselbergs was estimated using data from the Australian Gazetteer. This involved several stages. Firstly, latitude and longitude coordinates were extracted for all names containing the feature code Rock, Mt, Hill, and Range in southwest Australia bounded between −28° and −36° latitude and west of 127° longitude. These four feature codes are most commonly used to describe granite inselbergs (e.g., see Table 1). All locations were then imported into QGIS and intersected with a regolith layer to exclude those features not found on granite. For each of the 16 study inselbergs, the geographic distance to all other granite features was calculated in R and the number of inselbergs within a 20 km radius determined.

We extracted four preselected bioclimatic variables for each of the 16 inselbergs from the TERN e‐Mast facility which provides gridded bioclimatic information (0.01°) for the Australian continent using climate averages of monthly 1976–2005 data (Whitley et al., 2014). The four variables were mean temperature of the driest quarter (tdry), mean temperature of the wettest quarter (twet), precipitation of the driest quarter (pdry), and precipitation of the wettest quarter (pwet). These were chosen because they reflect variation in temperature and moisture during either optimal or limiting growth periods, and they have been shown by other studies to be strongly correlated with patterns of floristic diversity in the SWAFR (Gibson, Prober, Meissner, & Leeuwen, 2017; Jones et al., 2015).

### 2.4 Environmental data plot level

Average soil depth, based on five samples, was determined for each plot using a scaled (cm) soil probe (with a maximum range of 50 cm), following the approach of Houle and Phillips (1989).

To describe the topographic characteristics of plots, we obtained LiDAR data for the 16 outcrops by airplane using a Leica ALS50‐II scanner, flying in April 2011. Flight height was *ca*. 1,700–2,200 m, resulting in 0.63 points/m^2^, with ground (last) returns interpolated into a 2‐m grid using triangulation to create a digital elevation model (DEM). Horizontal accuracy and vertical accuracy were <0.35 and <0.15 m, respectively. Further details can be found in Schut et al. (2014). We derived raster surfaces for seven preselected topographic variables from the LiDAR‐based DEM for each of the 16 outcrops. These seven variables were aspect‐eastness, aspect‐northness, curvature, topographic position index (TPI), topographic wetness index (TWI), topographic ruggedness index (TRI), and saga wetness index (SWI). These variables were chosen because of their influence on the distribution of moisture across inselbergs. Aspect and curvature (second derivative of elevation) were calculated using tools available in the Spatial Analyst Toolbox of ArcGIS v 10.4 (ESRI, 2015). A curvature of 0 suggests the terrain is flat, whereas negative and positive values indicate convex terrain and concave terrain, respectively.

ModelBuilder (ESRI, 2015) was used to automate calculation of aspect‐eastness and aspect‐northness by taking the sine and cosine of aspect, respectively, after conversion to radians. Values range from −1 to 1 and represent the range for west to east (aspect‐eastness) or south to north (aspect‐northness) facing pixels (Samis & Eckert, 2009). We also automated calculation of the TWI and the TPI. TWI is based on the equation found in Gessler, Moore, McKenzie, and Ryan (1995) and is a steady‐state wetness index used to quantify topographic control on hydrological processes (Sørensen, Zinko, & Seibert, 2006). The TPI compares the elevation of each cell in a DEM to the mean elevation of a specified neighborhood around that cell and was calculated using a 100 × 100 m neighborhood size. Positive TPI values represent locations that are higher than the average of their neighborhood window (e.g., ridges), negative values are lower (e.g., valleys), and flat areas are close to 0 (Guisan, Weiss, & Weiss, 1999).

The TRI and SWI were calculated using SAGA software v 2.1.4 (Conrad et al., 2015). TRI, defined in Riley, De Gloria, and Elliott (1999), was used to quantify terrain heterogeneity. The SWI was used to supplement the TWI as it is reported to predict soil moisture in valley floors with small vertical distance to a channel more realistically (Boehner et al., 2002).

### 2.5 Statistical analyses

We investigated patterns of diversity in each of the three habitats at the plot and outcrop level. We did not compare patterns between the three habitats. At the plot scale, generalized linear mixed effects models (GLMMs) with Poisson distributions allowing for overdispersion (Lawless, 1987) were used to determine the relative correlations of soil depth, aspect‐eastness, aspect‐northness, curvature, TPI, TWI, TRI, and SWI with species richness in each of the three habitats. Separate models were fitted for each habitat treating inselberg as a random effect.

Models were fitted using the method recommended by Hosmer and Lemeshow (2000). First, we undertook a univariable analysis of each predictor variable with species richness and kept variables with *p* < 0.25. Second, we fitted a multivariable model using these retained variables and performed stepwise backward procedures, removing the least significant variable at each step until only significant variables (*p* < 0.05) remained. At each step in the backward elimination of variables, we compared the estimated coefficients in the reduced model from their respective values in the larger models to check that variables were not acting together. Changes in the magnitude of coefficients were acceptable, and no terms were added back to models. Finally, we added each variable initially omitted individually to the model obtained after backward selection, retaining any variable with *p* < 0.05 for inclusion in the final model. This is vital for identifying variables that by themselves are significantly related to the outcome, but that also make an important contribution to the presence of other variables.

At the inselberg scale, generalized linear models (GLMs) with a Poisson distribution allowing for overdispersion were used to determine the relative correlations of inselberg area, isolation, mean temperature driest quarter, mean temperature wettest quarter, precipitation driest quarter, and precipitation wettest quarter with species richness in each of the three habitats. Separate models were fitted for each habitat. We implemented GLMMs and GLMs in SAS using the GLIMMIX procedure (SAS, 2011). An approximate measure of goodness of fit for each model was determined from the squared correlation between the observed and predicted values.

We then used generalized dissimilarity modeling (GDM) to determine the relative importance of environmental dissimilarity and geographical distance with species turnover in each of the three habitats. GDM is a nonlinear matrix regression technique for analyzing spatial patterns in compositional dissimilarity (quantified with the Sorenson measure) between pairs of locations as a function of environmental dissimilarity and geographical distance (Ferrier, Manion, Elith, & Richardson, 2007; Fitzpatrick et al., 2013). GDM has advantages over classical linear matrix regression because it accommodates variation in the rate of compositional turnover (nonstationarity) at different points along a given gradient, and the curvilinear relationship between compositional dissimilarity and increasing environmental/geographical distance between sites (Ferrier et al., 2007; Fitzpatrick et al., 2013).

We implemented GDM with the *gdm* package in R (Manion, Lisk, Ferrier, Nieto‐Lugilde, & Fitzpatrick, 2016). We fitted a GDM using site‐by‐species and site‐by‐environment matrices for each of the three variables. We used the default of three I‐splines, with a backward elimination procedure retaining variables that made a significant contribution to explained deviance (*p* < 0.05) permuted 500 times at each step. We retained only significant variables in our final model.

We plotted the I‐spline for each significant predictor variable describing the relationship between species turnover and that variable. The slope of the I‐spline curves indicates the rate of turnover in species composition while the maximum height of each I‐spline curve represents the total amount of turnover associated with a variable gradient, holding all other variables constant. To quantify magnitude of turnover along each variable gradient and relative importance of that gradient in explaining species turnover in the model, we summed the three I‐spline coefficients (Fitzpatrick et al., 2013; Gibson et al., 2017).

To evaluate unique and shared contributions of climate variables, site variables, and geographic distance in explaining species turnover in the model, we used custom written R code to partition the deviance resulting from sets of four GDMs that used either climate variables, site variables, geographic distance, or all variables as predictor variables (Borcard, Legendre, & Drapeau, 1992; Gibson et al., 2017; Jones et al., 2015).

We investigated patterns of beta diversity at the inselberg scale. For each inselberg, we used the plot data to create species lists for each of the three habitat types. We calculated Baselga's multiple site dissimilarity measures (*β*_SOR_) for each of the three habitat types and the whole outcrop as well as their respective nestedness (*β*_NES_) and spatial turnover (*β*_SIM_) components using the *betapart* package in R (Baselga, 2010; Baselga & Orme, 2012).

## 3 RESULTS

### 3.1 Characteristics of the inselberg flora

We recorded 1,060 species from 92 families from the 478 plots. The Fabaceae (112 species), Myrtaceae (75), Asteraceae (77), Proteaceae (67), Cyperaceae (61), Orchidaceae (55), Poaceae (49), Asparagaceae (36), Ericaceae (34), and Stylidiaceae (32) were the ten most species‐rich families, but their relative abundance varied among the three habitats (Table 2).

**Table 2:** The ten most species‐rich families in the Southwest Australian Floristic Region compared to those for three habitats, herbaceous vegetation on outcrops (HO), woody vegetation on outcrops (WO), and woody vegetation at base of outcrops (WOB) on 16 granite inselbergs, sorted by the number of species (*n*) recorded in our plots per family

HO	*n*	WO	*n*	WOB	*n*	Total SWAFR^a^	*n*
Asteraceae	47	Myrtaceae	75	Fabaceae	79	Myrtaceae	1,436
Orchidaceae	31	Fabaceae	75	Myrtaceae	72	Fabaceae	1,156
Poaceae	24	Asteraceae	49	Asteraceae	51	Proteaceae	914
Cyperaceae	14	Orchidaceae	46	Proteaceae	49	Orchidaceae	422
Stylidiaceae	13	Proteaceae	45	Cyperaceae	43	Ericaceae	362
Droseraceae	13	Poaceae	40	Poaceae	37	Asteraceae	330
Centrolepidaceae	12	Cyperaceae	40	Orchidaceae	30	Cyperaceae	262
Asparagaceae	9	Ericaceae	27	Asparagaceae	28	Goodeniaceae	231
Araliaceae	8	Asparagaceae	22	Goodeniaceae	19	Stylidiaceae	227
Geraniaceae	7	Goodeniaceae	19	Ericaceae	18	Malvaceae	196

a Gioia and Hopper (2017) Appendix S3 https://naturemap.dpaw.wa.gov.au/resources/gh/index.html.

There were large numbers of rarely sampled species in our dataset, with 267 species (25.2%) recorded in a single plot across all plots and 544 species (51.3%) recorded in plots on a single inselberg (Table 3). Sixteen life‐form groups were recorded, with shrubs (37.4%), annual herbs (14%), perennial graminoids (12.3%), perennial herbs (11.3%), and perennial geophytes (7.2%) being most common (Table 3). Eight of the life‐form groups each included less than 2% of the taxa recorded.

**Table 3:** Life‐form spectra for the total flora from 478 plots on 16 granite inselbergs in the Southwest Australian Floristic Region, and species occurring in only one single plot (on all inselbergs)or to plots on a single inselberg

Life‐form	No species	Total flora (%)	Single plot	Single plot (%)	Single inselberg	Single inselberg (%)
Annual fern	1	0.1	1	0.4	1	0.2
Annual graminoid	48	4.5	8	3.0	13	2.4
Annual herb	148	14.0	41	15.4	62	11.4
Annual succulent herb	12	1.1	2	0.7	3	0.6
Perennial climber/epiphyte	26	2.5	5	1.9	10	1.8
Perennial climber/epiphyte ‐parasite	5	0.5	0	0.0	0	0.0
Perennial fern	13	1.2	3	1.1	6	1.1
Perennial graminoid	130	12.3	29	10.9	70	12.9
Perennial herb	120	11.3	26	9.7	62	11.4
Perennial herb–carnivore	4	0.4	1	0.4	4	0.7
Perennial herb geophyte	77	7.3	17	6.4	31	5.7
Perennial herb geophyte–carnivore	14	1.3	3	1.1	7	1.3
Perennial succulent herb	2	0.2	0	0.0	0	0.0
Shrub	396	37.4	120	44.9	243	44.7
Tree	54	5.1	8	3.0	27	5.0
Shrub or tree parasite	10	0.9	3	1.1	5	0.9
*N*	1,060		267		544	

Alien species made up approximately 7% of the total flora with annual herbs being the most common life‐form (39 species, 26% of annual herbs) followed by annual graminoids (19 species, 40% of annual graminoids), perennial herbs (nine species, 8% of perennial herbs), annual succulent herbs (three species, 25% of annual succulent herbs), and perennial graminoids (two species, <1% of perennial graminoids).

### 3.2 Diversity of the inselberg flora

At the plot level, local variables were significantly correlated (*p* < 0.05) with species richness in HO and WO plots. Mean species richness in HO plots ranged from 7.8 to 18.0 species per plot and increased with increasing saga wetness index (*t*_15,148_ = 4.94, *p* < 0.001, *r* ^2^ = 0.49). Mean species richness in WO plots ranged from 13.5 to 26.3 species per plot and increased with soil depth (*t*_15,151_ = 3.3, *p* < 0.01) and decreased with increasing TPI (*t*_15,151_ = −2.2, *p* < 0.05) with the model including both variables having an *r* ^2^ = 0.29. Mean species richness in WOB habitats varied from 14.3 species to 57.0 species per plot but was not correlated with any of the soil depth or topographic variables.

At the outcrop level, species richness in HO plots was relatively constant across inselbergs and not correlated with outcrop area, isolation, or any of the four bioclimatic variables. Total species richness in WO and WOB plots was not correlated with inselberg area or isolation, nor with three of the bioclimatic variables. However, species richness increased significantly with precipitation of the driest quarter for WO plots (Wald $\chi_{1,14}^{2}$ = 11.83, *p* < 0.001, *r* ^2^ = 0.45) and decreased significantly with precipitation of the driest quarter for WOB plots (Wald $\chi_{1,14}^{2}$ = 7.28, *p* < 0.01, *r* ^2^ = 0.31).

### 3.3 Contribution of environment and geographic space to species turnover

**Figure 3:**
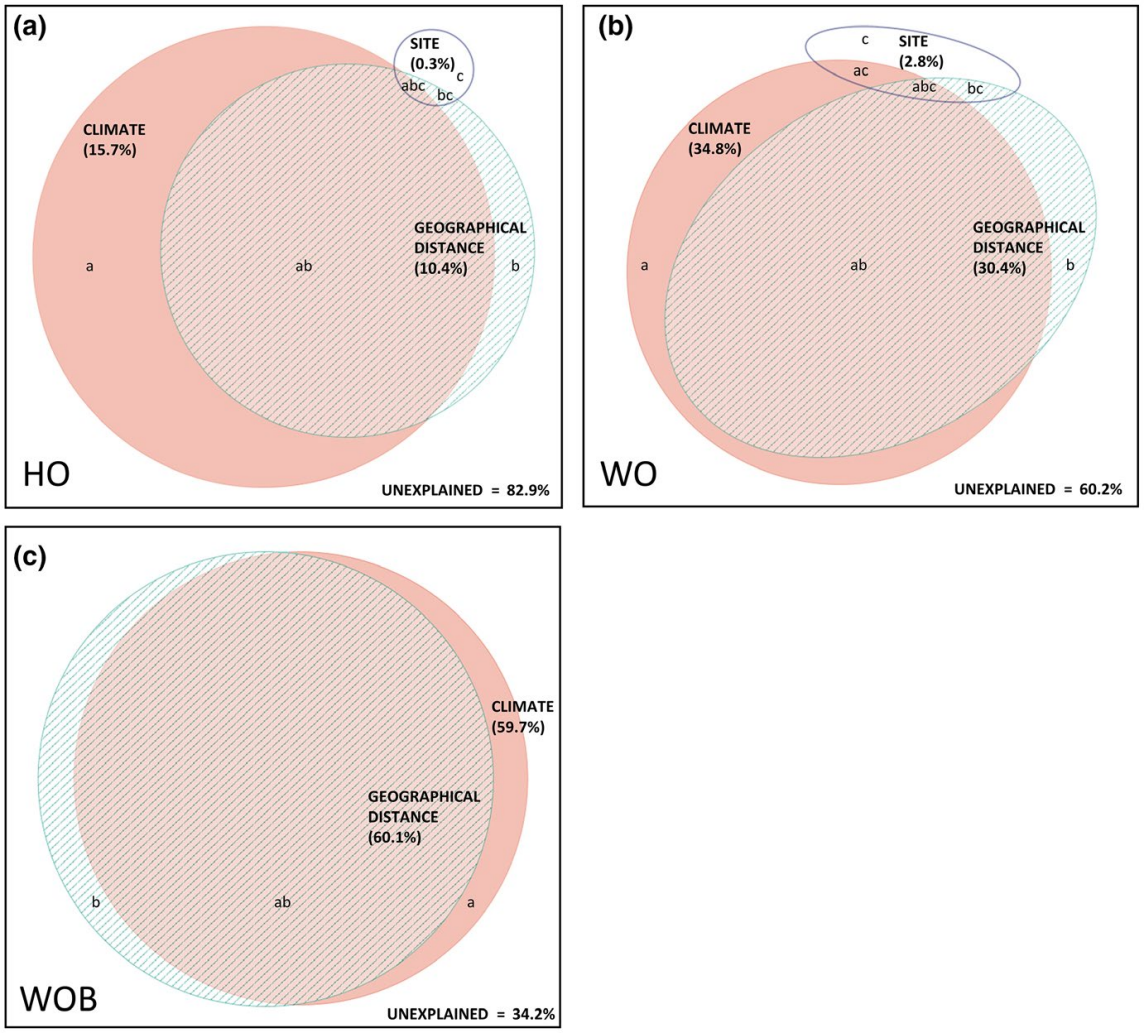
Partitioning of generalized dissimilarity model deviance explained in plant species turnover on 16 granite inselbergs across the Southwest Australian Floristic Region for three habitats: (a) herbaceous vegetation of soil‐filled depressions on the outcrop (HO); (b) woody vegetation of soil‐filled depressions on the outcrop (WO); and (c) woody vegetation on deeper soils at the base of outcrops (WOB). Three sets of explanatory variables were used: climatic, geographical distance, and site variables. For full details of the unique and shared contributions to explained deviance, see Table 5. The Venn diagram was drawn in eulerAPE 3 (Micallef & Rodgers, 2014)

Four variables were retained as predictors of species turnover in the GDM for HO plots (Table 4). Precipitation of the driest quarter was relatively the most important variable followed by precipitation of the wettest quarter, interplot geographic distance, and aspect‐northness. The four variables accounted for 17.1% of the total explained model deviance (Figure 3, Table 5). Six variables were retained as predictors of species turnover in the GDM for WO plots (Table 4). Interplot geographic distance was relatively the most important variable followed by mean temperature of the driest quarter, precipitation of the driest quarter, precipitation of the wettest quarter, soil depth, and topographic ruggedness index. The six variables accounted for 39.9% of the total explained model deviance (Figure 3, Table 5). Three variables were retained as predictors of species turnover in the GDM for WOB plots (Table 4). Interplot geographic distance was relatively the most important variable followed by precipitation of the wettest quarter and mean temperature of the driest quarter. The three variables accounted for 65.8% of the total explained model deviance (Figure 3, Table 5).

**Table 4:** Relative importance of the significant predictor variables used for modeling the compositional dissimilarity of plant communities in three habitats across 16 granite inselbergs in the Southwest Australian Floristic Region

Gradient	HO	WO	WOB
Interplot geographic distance	0.34	1.21	2.02
Mean temperature wettest quarter	—	—	—
Mean temperature driest quarter	—	0.91	1.81
Precipitation wettest quarter	0.47	0.86	1.40
Precipitation driest quarter	0.78	1.11	—
Soil depth	—	0.46	–
Aspect‐eastness	—	—	—
Aspect‐northness	0.15	—	—
Curvature	—	—	—
Topographic position index	—	—	—
Topographic wetness index	—	—	—
Topographic ruggedness index	—	0.39	—
Saga wetness index	—	—	—

Note

**Figure 4:**
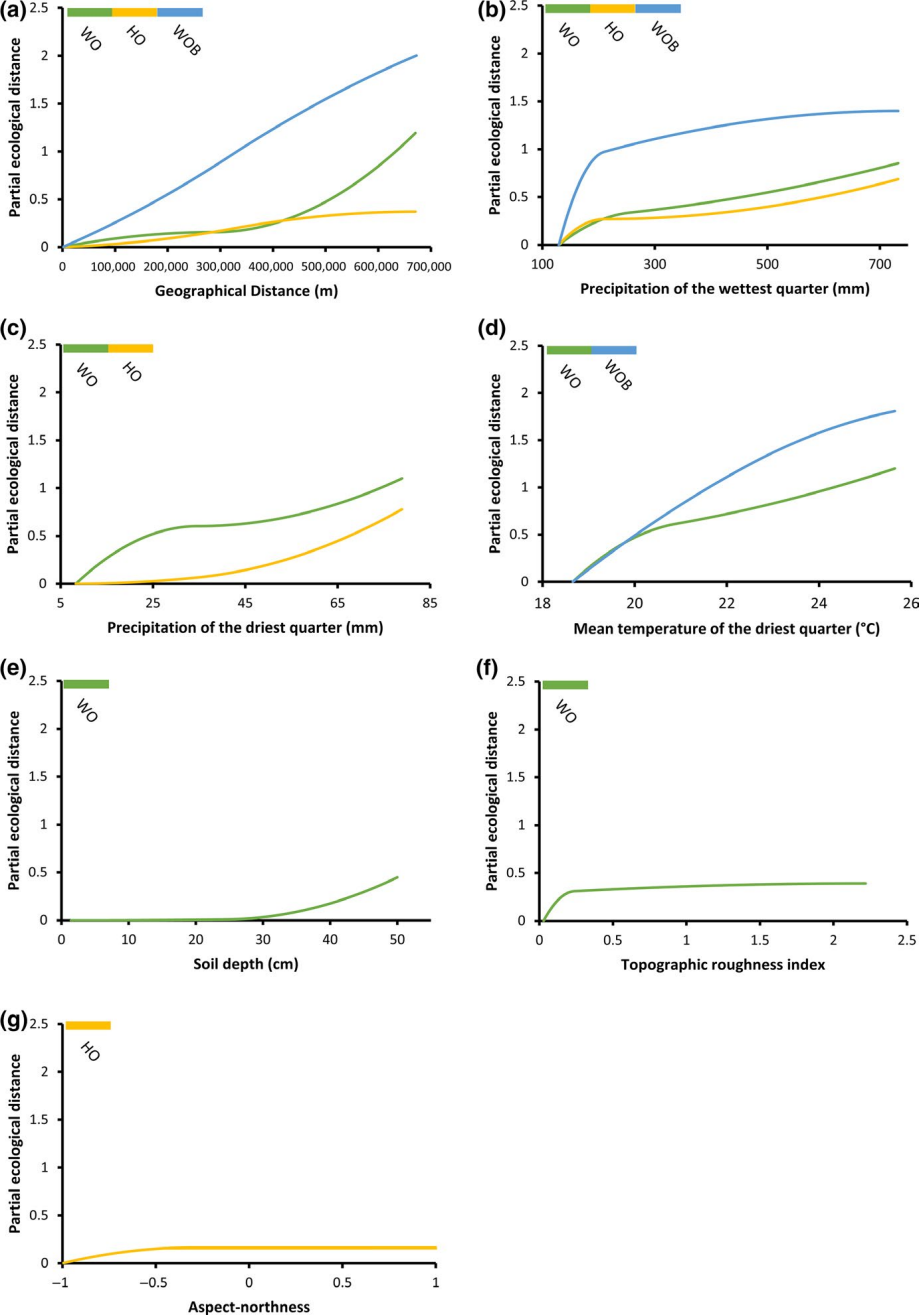
Generalized dissimilarity model‐fitted I‐splines for variables found to be significantly associated with patterns of beta diversity across 16 granite outcrops in the Southwest Australian Floristic Region divided into three habitat types: herbaceous vegetation of soil‐filled depressions on the outcrop (HO); woody vegetation of soil‐filled depressions on the outcrop (WO); and woody vegetation on deeper soils at the base of outcrops (WOB). The maximum height reached by each curve indicates the total amount of compositional turnover associated with that variable, holding other variables constant and its shape indicates the rate of compositional turnover variation along the gradient

Habitats are herbaceous vegetation of soil‐filled depressions on the outcrop (HO); woody vegetation of soil‐filled depressions on the outcrop (WO); and woody vegetation on deeper soils at the base of outcrops (WOB). Relative importance is determined by summing the coefficients of the I‐splines (each I‐spline has three coefficients) from generalized dissimilarity modeling (see Figure 4).

**Table 5:** Generalized dissimilarity model deviance in plant species turnover on 16 granite outcrops explained by selected sets of variables (climate, geographical distance, and site) partitioned into corresponding unique (a,b,c) and shared (ab, ab, bc, abc) contributions as annotated on Figure 3

Explanatory set	Habitat		
	HO	WO	WOB
Climate (a)	6.5	7.2	5.7
Geographical distance (b)	1.0	2.9	6.1
Site (c)	0.35	1.8	0.0
Climate ∩ geographical distance (ab)	9.3	27.0	54
Climate ∩ site (ac)	0.0	0.4	0.0
Site ∩ GD (bc)	0.05	0.3	0.0
Climate ∩ site ∩ geographical distance (abc)	0.05	0.3	0.0
Total (climate ∪ site ∪ GD)	17.2	39.9	65.8

Note

HO, herbaceous vegetation of soil‐filled depressions on the outcrop (HO); WO, woody vegetation of soil‐filled depressions on the outcrop (WO); WOB, woody vegetation on deeper soils at the base of outcrops (WOB).

The I‐spline fitted functions describing the magnitude and rate of compositional differences in the three habitats were nonlinear for most variables (Figure 4), with rates of turnover varying with position along the aridity gradient, being greatest at low levels of precipitation of the wettest quarter (Figure 4b). Additionally, in WO plots rates of species turnover increased with soil depth (Figure 4e) and low values of TRI (Figure 4f), while in HO plots rates of species turnover increased at a greater rate in plots with a more southerly aspect (Figure 4g).

### 3.4 Relative contribution of spatial turnover and nestedness to compositional differences

Beta diversity, as measured by Baselga's multiple site dissimilarity measure (*β*_SOR_), ranged from 0.892 to 0.935 for the three habitat types on the inselbergs. This was almost entirely due to spatial species turnover (*β*_SIM_ range 0.875–0.910) with a very low component due to nestedness (*β*_NES_ 0.017–0.025; Table 6). A similar pattern occurred for the whole outcrop flora.

**Table 6:** Baselga's multiple site dissimilarity measure (*β*_SOR_) and its components related to species replacement (*β*_SIM_) and nestedness (*β*_NES_) across granite inselbergs in the Southwest Australian Floristic Region

	*β*_SIM_	*β*_NES_	*β*_SOR_
HO	0.875	0.017	0.892
WO	0.910	0.017	0.927
WOB	0.910	0.025	0.935

Note

Values are given for plots from three habitat types on the inselbergs, herbaceous vegetation of soil‐filled depressions on the outcrop (HO); woody vegetation of soil‐filled depressions on the outcrop (WO); and woody vegetation on deeper soils at the base of outcrops (WOB).

## 4 DISCUSSION

Our analysis of patterns of species diversity in three granite inselberg plant communities in the SWAFR confirms our expectation that species richness and variation in floristic composition are generally correlated with regional‐scale climate gradients reinforced by local‐scale variables affecting aridity. Further, compositional dissimilarity (beta diversity) increases with environmental and geographic distance. For all three habitats, species replacement and not nestedness (species loss) is responsible for variation in species composition. The high species replacement on granite inselbergs in the SWAFR mirror patterns found on other OCBIL landscapes in the SWAFR flora (Gibson, Meissner, Markey, & Thompson, 2012; Gibson et al., 2017). These patterns indicate that effective conservation strategies are best focused on protecting multiple inselbergs across the entire climate gradient of the region to protect the high level of biodiversity in the region brought about by spatial turnover in species composition.

### 4.1 Species richness, compositional characteristics, and environmental gradients of the inselberg flora

Our investigation of floristic diversity on granite inselbergs in SWAFR recorded large numbers of species, with many recorded at low frequencies. Over 25% of taxa were restricted to a single plot and 51% to a single inselberg. Our results concur with those of Porembski and Brown (1995) for granite inselbergs in tropical western central Africa where 75% of all species occurred on fewer than 5 (out of 25) inselbergs, with 37% of species restricted to a single inselberg. High numbers of rare species have also been recognized on Banded Iron Formation (BIF) inselbergs (Gibson et al., 2012) and in other vegetation types in the SWAFR (e.g., Gibson et al., 2017; Keighery, Gibson, van Leeuwen, Lyons, & Patrick, 2007; Wardell‐Johnson & Williams, 1996). Analysis of evolutionary patterns in floras of granite and BIF inselbergs in the SWAFR shows that these landscape features are areas where species can persist through harsh climatic conditions. This leads to both diversity within, and differentiation among, inselbergs at both species and genetic levels (Byrne & Hopper, 2008; Byrne et al., 2018; Tapper et al., 2014a, 2014b).

Our analyses show that aridity gradients associated with local site variables were strongly correlated with species richness in the two habitats (HO and WO) restricted to the inselberg surface. We found no correlation of local site variables on species richness in plant communities at the base of inselbergs (WOB) perhaps because these sites are topographically less complex, are typically areas where water drains to, and soils are frequently >50 cm deep. However, at the outcrop level a regional aridity gradient was correlated with the number of species found in WOB habitats. A negative correlation between the number of species and precipitation of the driest quarter for WOB plots contrasts with that from WO plots. In our study, tall forest dominated by *Eucalyptus diversicolor* (karri) surrounds the base of granite inselbergs in the highest rainfall zone (i.e., Mt Chudalup and Mt Frankland; Figure 1), whereas low open woodlands are common around the base of granite inselbergs in more arid regions. These forests are of higher biomass and generally lower diversity compared to other vegetation types in the SWAFR (Wardell‐Johnson & Williams, 1996). There are multiple evolutionary and ecological factors that may contribute to low species diversity in the tall forests of the SWAFR, particularly the relatively small area of tall open forest (204,000 ha pre‐1750; Wardell‐Johnson, Neldner, & Balmer, 2017) and loss of virtually all rainforest taxa with aridification in the Neogene (Byrne et al., 2011; Dodson & McPhail, 2004).

Generally, tall open eucalypt forest is restricted to freely drained sites in the SWAFR receiving over 1,000 mm MAR and at least 25 mm in the driest month (Churchill, 1968). However, the range of the karri forest is extended eastwards into drier zones with a significant outlier occurring at the base of one of our study sites in the Porongurup Range (Churchill, 1968). Here, the large granite domes intercept moisture‐laden prevailing winds year‐round from the south coast creating an orographic effect (Keppel et al., 2017). Run‐off from the outcrops focuses water at the base of the inselberg creating a localized increase in water availability that is enough to support karri forest. A similar effect was observed in the Namib Desert where Burke (2002) recorded species from neighboring higher rainfall areas at the base of granite inselbergs.

Porembski et al. (2000) found a positive correlation between granite inselberg area and the total number of vascular plant species for outcrops in the Cȏte d'Ivoire. They attributed this to island biogeographic factors associated with an increase in the diversity of habitats and a reduced risk of local extinction due to populations being larger on bigger inselbergs. In our study, we examined patterns of species diversity and their correlates within each of three granite habitats. We found no correlation between inselberg area or isolation and species richness for the three habitats. Ornduff (1987) in an early study of SWAFR granite inselbergs also found no correlation between inselberg area and species richness in the equivalent of our HO habitat. Ornduff (1987) suggested that the simplest explanation for this finding is that only a small number of vascular plant species can successfully occupy a distinctive and limited array of microsites within a habitat and that these occur independent of inselberg size. More recently, Hopper (2009) suggested that plant species on OCBILs, such as the granite inselbergs of the SWAFR, are more likely to have undergone natural selection for genetic, cytogenetic, or phenotypic adaptations that conserve heterozygosity or maintain viability in the face of inbreeding due to small population size. This would thereby reduce their risk of extinction as exemplified by the granite‐endemic herb *Isotoma petraea* (Bussell, Waycott, Chappill, & James, 2002; James, 1965) and *E. caesia* (Bezemer, Krauss, Phillips, Roberts, & Hopper, 2016; Byrne & Hopper, 2008). Additionally, microrefugia on outcrops may also allow species to migrate “in situ” around an outcrop as conditions change and buffer species against local extinction (Tapper et al., 2014a, 2014b). Consequently, inselberg area and population size may be less important for persistence in these landscapes.

### 4.2 Contribution of environment and geographic space to compositional differences

Generalized dissimilarity modeling showed a strong relationship between species turnover and aridity in the three habitats. Climate variables reinforced by local site variables are important correlates of species turnover in HO and WOB plots, but only climate variables were associated with species turnover in WOB plots. Similarly, a strong influence of climate gradients on species turnover was reported for granite inselbergs in tropical western central Africa (Parmentier et al., 2005) and for BIF inselbergs in southwestern Australia (Gibson et al., 2012). Interplot geographic distance, which is often interpreted as a surrogate for dispersal limitation, made small independent contributions to species turnover in the three habitats, but overlapped with climate because the species turnover and climate gradients varied at similar scales. Jones et al. (2015) also reported that interplot distance overlapped with climate for shrublands and woodlands in the Transitional Rainfall Zone of the SWAFR.

It is well established that soil depth has a strong influence on the physiognomy and floristic composition of granite inselberg vegetation (Burbanck & Platt, 1964; Schut et al., 2014), and that both physiological tolerances and competitive interactions contribute to this pattern (Houle & Phillips, 1989; Poot, Hopper, & van Diggelen, 2012). In our study, we investigated three plant communities occupying different parts of the soil depth gradient on or around inselbergs and found that soil depth had an impact on the species richness and composition in woody vegetation in soil‐filled depressions, but not in herbaceous vegetation in soil‐filled depressions.

The influences of other local site variables associated with topographic variation, that also affect plant growth and reproduction, such as water flows, have received less attention than soil depth despite their potential importance. For example, Keppel et al. (2017) revealed the significant correlations of elevation and topographic variables (insolation, curvature, and aspect) at the local scale with the distribution of two closely related granite‐endemic herb species. We found that at least for herbaceous and woody vegetation of soil‐filled depressions, local site variables played a smaller but reinforcing role to regional aridity gradients influencing species richness and composition.

Collectively, the results of our study show similar patterns of spatial variation in floristic composition in granite inselberg plant communities in the Mediterranean climate region of the SWAFR to those reported for granite inselbergs in tropical and arid regions (Burke, 2001; Parmentier et al., 2005; Porembski et al., 1998). From their analysis, Parmentier et al. (2005) considered the best explanation for the spatial patterns in floristic composition was impacts of different processes at local and regional scales. At a local scale, species composition is best explained by soil depth creating a diversity of ecological niches, whereas at a regional scale, these niches are occupied by a variety of species depending on the composition of the local species pool (Parmentier et al., 2005). Our study similarly demonstrates the influence of soil depth on the species composition of granite inselberg plant communities, but also shows that other topographic variables affecting aridity are influential.

### 4.3 Relative contribution of spatial turnover and nestedness to compositional differences

Beta diversity, as measured by a multiple site dissimilarity measure (Baselga, 2010), was uniformly high for the three habitat types, and almost entirely due to spatial species turnover with a very low component due to nestedness. Early descriptive studies of granite inselberg floras in the SWAFR allude to this high turnover. For example, Ornduff (1987) noted substantial dissimilarity in the composition of herbaceous vegetation among inselbergs within the same region, with outcrops less than 1 km apart having quite different floras. Hopper et al. (1997) also observed similar trends for orchids. While not being directly comparable with our multiple site measure of beta diversity, it is worth noting that Hunter (2000, 2004) measured both high levels of spatial structure in species richness, and low levels of nestedness in the composition of granite inselberg floras on the New England Batholith in temperate eastern Australia. Similar patterns in multiple site measures of beta diversity have also been measured for other OCBIL landscapes in the SWAFR, including BIF inselberg and lateritic sandplain floras (Gibson et al., 2012, 2017). In the latter, spatial species turnover increased rapidly to 10 km, leveled off at about 50 km, and then increased slowly until maximum extent of *ca*. 870 km was reached (Gibson et al., 2017). Thus, the factors that drive species turnover in the sandplain floras in the SWAFR also seem to operate in inselberg plant communities.

The local species pool on a granite inselberg may reflect the interplay between regional processes, such as species formation and geographic dispersal, and local processes such as the physiological tolerances of species, competition between them, and stochastic variation in the physical environment. Regional processes add species to communities, while local processes promote local extinction (Ricklefs, 1987). In the SWAFR, differences in species pools across granite inselbergs likely result from regional processes, such as climate gradients and the geoclimatic history of the region, as well as local processes of limited dispersal and stochasticity, that together affect the balance of speciation and extinction (Hopper, 2009; Hopper et al., 1997; Hopper & Gioia, 2004).

In our GDMs, we were unable to separate the influence of climate gradients from interplot geographic distance, which is considered a surrogate for dispersal limitation. However, genetic and phylogeographic studies of endemic species from two of the most abundant life‐forms on SWAFR inselbergs, shrubs, and perennial herbs, show genetic signals of prolonged isolation, persistence, and divergence of populations on specific granite inselbergs through Pleistocene climate cycles (Byrne & Hopper, 2008; Tapper et al., 2014a, 2014b; Yates et al., 2007). Importantly, these results indicate that both geographically widely distributed and rare species are maintained on inselbergs through persistence, most likely in microrefugia, and not by long‐distance seed dispersal from source populations. It is therefore highly likely that dispersal limitation, at least for many perennial shrubs and herbs, is a significant factor in species turnover on SWAFR granite inselbergs. Further research is needed to understand whether dispersal is limiting for other life‐forms, particularly widespread annual herbs and grasses, with seed morphologies assisting wind dispersal.

Furthermore, studies of plant population and community dynamics on granite inselbergs in the SWAFR also show that stochastic processes like fire and extreme drought can result in local population extirpation, and strongly influence the distributions of species and community composition (Hunter, 2017; Yates et al., 2003, 2007). In addition, recent evidence indicates that a relatively buffered Pleistocene climate and near‐quiescent Cenozoic topography in the SWAFR may have reduced the intensity of extinctions associated with Pleistocene climate cycles and promoted the persistence of species‐rich pre‐Pliocene clades (Byrne et al., 2014; Cowling et al., 2015; Hopper, 2009; Hopper, Silveira, & Fiedler, 2016; Sniderman, Jordan, & Cowling, 2013).

### 4.4 Conservation implications

Compared to the SWAFR flora, the region's granite flora has a higher richness of taxa in families containing annual and perennial grasses (Poaceae), annual herbs (e.g., Asteraceae), perennial herbs (e.g., Asparagaceae), and geophytes (e.g., Droseraceae, Orchidaceae). Thus, our results confirm the importance of granite inselbergs for conservation of the region's extraordinary plant diversity (Hopper et al., 1997).

Of the 7% of the flora recorded that were alien, the majority (66%) were annuals. Some habitats, such as seasonally bare HO habitats, may be prone to weed establishment due to high annual seed rain from surrounding cleared lands. Nevertheless, our study does suggest capacity for the local flora to persist within relatively small reserves despite being surrounded by agriculture. Interestingly, HO plots included annual weeds regardless of distance to cleared land, likely due to high dispersal features of many annual weed species.

Spatial species turnover accounts for almost all the beta diversity across granite inselbergs in the SWAFR. Effective conservation strategies should therefore focus on protecting multiple inselbergs across the entire climate gradient of the region, rather than on a few inselbergs or on those with greatest species richness (Baselga, 2010). Fortunately, many inselbergs have always been highly valued as water‐sources, including during Western Australia's agricultural development. Further, they were unsuitable for agriculture and thus were spared from vegetation clearing. Many granite inselbergs occur in conservation reserves, although they do occur across a variety of other tenures. Thus, effective conservation of their rich plant diversity will rely on continuing “off‐reserve” initiatives that minimize disturbance. Measurement of species composition and turnover on granite inselbergs and their environmental and spatial correlates provides a basis for conservation planning at both local and regional scales across this global biodiversity hot spot, where conservation of many inselbergs is the most appropriate strategy to facilitate ongoing persistence of these biodiverse species‐rich communities.

## CONFLICT OF INTEREST

There are no conflicts of interest.

## AUTHOR CONTRIBUTIONS

GWJ, MB, SDH, and CJY gained funding for the study. All authors conceived the ideas and contributed to design of the methodology. CJY, GWJ, GK, TR, and AGTS collected the data. CJY and TR analyzed the data. CJY led the manuscript writing, and all remaining authors contributed to draft revisions.

## Data Availability

Data are available through the Dryad Digital Repository https://datadryad.org/ (https://doi.org/10.5061/dryad.35r27v6).
